# The Siamese Twins

**Published:** 1874-02

**Authors:** 


Amongst the dimly-recalled remembrances of our childhood comes to mind the fact of having dined at a farm-house in Columbia county, Georgia, with two dusky sons of a far-off Indian clime, so strangely linked to each other as to require that a bench be brought in for their accommodation at table. To-day the telegraphic dispatches bring us the intelligence of the death, on Saturday 17th January, of the Siamese Twins, Chang and Eng, at their home near Mount Airy, Surrey county, North Carolina. The meagre particulars we gather state that Chang was paralyzed last fall, since which time he had been fretful, very much debilitated, and strongly addicted to drink, as a means of alleviating his sufferings. He had been quite feeble for several days—indeed, so much so as to confine both brothers to bed; but their illness was not so great as to cause any anticipation of the catastrophe that was to follow. On Friday night they retired to bed as usual, but during the night Chang became worse, and expired suddenly at about four o'clock on Saturday morning. As soon as it was discovered that Chang was dead, Eng became so terribly shocked that he raved wildly for awhile, exclaiming, "I suppose I must die, too!" and at times exhibiting signs of great mental aberration. In two hours from the death of Chang, Eng breathed his last.

The twins were born in 1811, and were, therefore, in their sixty-third year.

The proposal of a *post-mortem* examination of the bond of union between the brothers was rejected by the families, and, it is said, was forbidden in their will. The medical profession, the world over, will deeply regret that a full and accurate necropsy could not be made. We learn that Professor Pancoast, of the Jefferson Medical College, through Mayor Stokely, of Philadelphia, has telegraphed the Mayor of Greensboro, North Carolina, asking for an examination in the interest of science.

P. S.—Since the above has gone into type, we learn by telegram that an autopsy is yet to be held by Drs. Pancoast and Allen, of Philadelphia, in conjunction with Dr. Hollingsworth, the attending physician.

